# RNA activation in ticks

**DOI:** 10.1038/s41598-023-36523-4

**Published:** 2023-06-08

**Authors:** Kofi Dadzie Kwofie, Emmanuel Pacia Hernandez, Anisuzzaman, Hayato Kawada, Yuki Koike, Sana Sasaki, Takahiro Inoue, Kei Jimbo, Fusako Mikami, Danielle Ladzekpo, Rika Umemiya-Shirafuji, Kayoko Yamaji, Tetsuya Tanaka, Makoto Matsubayashi, Md Abdul Alim, Samuel Kweku Dadzie, Shiroh Iwanaga, Naotoshi Tsuji, Takeshi Hatta

**Affiliations:** 1 Department of Parasitology and Tropical Medicine, Kitasato University School of Medicine, Sagamihara, Kanagawa 252-0374 Japan; 2 Department of Parasitology, Noguchi Memorial Institute for Medical Research, College of Health Sciences, University of Ghana, P.O. Box LG 581, Legon, Accra, Ghana; 3 Department of Veterinary Paraclinical Sciences, College of Veterinary Medicine, University of the Philippines at Los Baños, College, 4031 Laguna, Philippines; 4 Department of Parasitology, Faculty of Veterinary Science, Bangladesh Agricultural University, Mymensingh, 2202 Bangladesh; 5 Department of Molecular and Cellular Parasitology, Graduate School of Medical Sciences, Kitasato University, Sagamihara, Kanagawa 252-0374 Japan; 6 Department of Environmental Parasitology, Tokyo Medical and Dental University, Bunkyo-ku, Tokyo, 113-8510 Japan; 7 National Research Center for Protozoan Diseases, Obihiro University of Agriculture and Veterinary Medicine, Obihiro, Hokkaido 080-8555 Japan; 8 Department of Tropical Medicine and Center for Medical Entomology, The Jikei University School of Medicine, Minato-ku, Tokyo, 105-8461 Japan; 9 Laboratory of Infectious Diseases, Joint Faculty of Veterinary Medicine, Kagoshima University, Kagoshima, 890-0065 Japan; 10 Department of Veterinary Immunology, Graduate School of Veterinary Sciences, Osaka Metropolitan University, Izumisano, Osaka 598-8531 Japan; 11 Department of Molecular Protozoology, Research Institute for Microbial Diseases, Osaka University, Yamadaoka, Suita, Osaka 565-0871 Japan; 12 Center for Infectious Disease Education and Research (CIDER), Osaka University, Yamadaoka, Suita, Osaka 565-0871 Japan

**Keywords:** Parasite biology, RNAi

## Abstract

RNA activation (RNAa) is a burgeoning area of research in which double-stranded RNAs (dsRNAs) or small activating RNAs mediate the upregulation of specific genes by targeting the promoter sequence and/or AU-rich elements in the 3′- untranslated region (3’-UTR) of mRNA molecules. So far, studies on the phenomenon have been limited to mammals, plants, bacteria, *Caenorhabditis elegans,* and recently, *Aedes aegypti*. However, it is yet to be applied in other arthropods, including ticks, despite the ubiquitous presence of argonaute 2 protein, which is an indispensable requirement for the formation of RNA-induced transcriptional activation complex to enable a dsRNA-mediated gene activation. In this study, we demonstrated for the first time the possible presence of RNAa phenomenon in the tick vector, *Haemaphysalis longicornis* (Asian longhorned tick). We targeted the 3ʹ-UTR of a novel endochitinase-like gene (HlemCHT) identified previously in *H. longicornis* eggs for dsRNA-mediated gene activation. Our results showed an increased gene expression in eggs of *H. longicornis* endochitinase-dsRNA-injected (dsHlemCHT) ticks on day-13 post-oviposition. Furthermore, we observed that eggs of dsHlemCHT ticks exhibited relatively early egg development and hatching, suggesting a dsRNA-mediated activation of the HlemCHT gene in the eggs. This is the first attempt to provide evidence of RNAa in ticks. Although further studies are required to elucidate the detailed mechanism by which RNAa occurs in ticks, the outcome of this study provides new opportunities for the use of RNAa as a gene overexpression tool in future studies on tick biology, to reduce the global burden of ticks and tick-borne diseases.

## Introduction

The manipulation of gene expression is instrumental to understanding disease mechanisms and developing novel therapeutics^1–8^. In the last decade, this technology has focused mainly on gene silencing through the process of RNA interference (RNAi), based on the primary understanding of noncoding small RNAs (ncRNAs) such as double-stranded RNAs (dsRNAs) and short interfering RNAs (siRNAs)^4,5,9^, where gene function is assessed by the suppression of the gene expression. Recent reports have revealed a new role for ncRNAs in a phenomenon known as RNA activation (RNAa)^2,10–13^. In many genes, ncRNAs are reported to overlap the 3′- untranslated region (3ʹ-UTR) which plays a pivotal role in cellular regulation and disease pathology^14^. Although the role of terminus-associated ncRNAs is not understood, their association with the 3ʹ-UTR suggests that they may affect gene regulation^15^. It was observed that ncRNAs targeting the 3ʹ-UTR of the PR gene caused either transcriptional gene silencing or activation by interacting with an overlapping noncoding sense transcript^16^. In RNAa, dsRNAs, also referred to as small activating RNAs (saRNAs), target the promoter sequence and/or AU-rich elements in 3ʹ-UTR of mRNA molecules and regulate gene expression at the transcriptional/epigenetic level^16^. It provides a tool for the specific activation of endogenous and exogenous genes^17^. Even though both RNAi and RNAa utilize ncRNAs, there are key differences. While siRNAs are loaded to argonaute (AGO) proteins 1–4 in RNAi^1^, saRNAs specifically bind to AGO 2 protein, which is an indispensable requirement for dsRNA-mediated gene activation^1,11,18,19^. In addition, unlike RNAi, RNAa occurs in the nucleus of cells, where transcriptional elements are recruited to upregulate gene expression^11^. The recruitment of these elements results in the formation of RNA-induced transcriptional activation (RITA) complex^18,19^, which either binds directly to the DNA or binds to a newly formed transcript in the region^20–22^.

RNAa was first discovered, when saRNAs designed to have complementary sequences to the promoter regions of E-cadherin, p21, and vascular endothelial growth factor (VEGF), were observed to mediate sequence-specific mRNA transcription activation of the respective targeted genes in mammalian cells^11^. Subsequently, the phenomenon has been applied to several other cells of mammals, plants, bacteria, and *Caenorhabditis elegans*^10,13,23–27^ suggesting that this pathway is highly regulated and evolutionarily conserved^10^. Recently, the presence of RNAa has been demonstrated and reported in *Aedes aegypti,* the arthropod vector of Dengue fever^17^. Interestingly, there has not been any report of RNAa in any other arthropod vector, despite the ubiquitous presence of AGO 2 in other arthropods^28^, including ticks^29,30^.

Arthropod chitinases have been shown to be essential components in the hydrolysis of chitin, present in the exoskeleton and peritrophic membrane (PM) in the gut of arthropods, to allow degradation of the cuticle and development during the immature stages^31,32^. In developmental events such as, embryogenesis, egg hatching, blood feeding, engorgement, and molting at various tick stages, chitinolytic enzymes such as chitinase are essential to remove old cuticle and allow synthesis of new cuticle for continued growth and development. Over the years, chitinases have been identified in different ticks species, including *Haemaphysalis longicornis* (Asian longhorned tick)^31–34^.

*H. longicornis* is a three-host tick, native to countries within eastern Asia, such as Korea, Japan and China^32,35–37^. It is also a highly invasive tick species, well established as exotic species in Australia, New Zealand and recently, in the United States of America^32,35–37^. In addition, it is a zoonotic vector of *Dabie bandavirus*^38^, *Anaplasma*, *Babesia*, *Borrelia*, *Ehrlichia* and *Rickettsia* pathogen species^23,35–37^. Their vectoral capacity is greatly influenced by their high reproductive potential in laying several thousand eggs, which hatch between 21 and 25 days post-oviposition (dpo) at a temperature of 25 °C^39^. Chemical acaricides have been the primary control method. However, limitations in using acaricides have raised suggestions on applying gene expression manipulation technologies such as RNAi, CRISPR-Cas, and RNAa in the control of ticks^40–42^. We recently identified a novel endochitinase-like (HlemCHT) gene tag, S03044-17L21 from the expressed sequence tag (EST) database derived from *H. longicornis* egg-specific complementary DNA (cDNA) library^43^**.** In trying to elucidate the function of this newly identified gene with RNAi, we coincidentally encountered the phenomenon of RNAa in the tick egg. In this study, we therefore report our findings and the potential use of RNAa in vector control. This is the first attempted evidence of RNAa in ticks.

## Results

### Sequence analysis of the full-length HlemCHT

**Figure 1:**
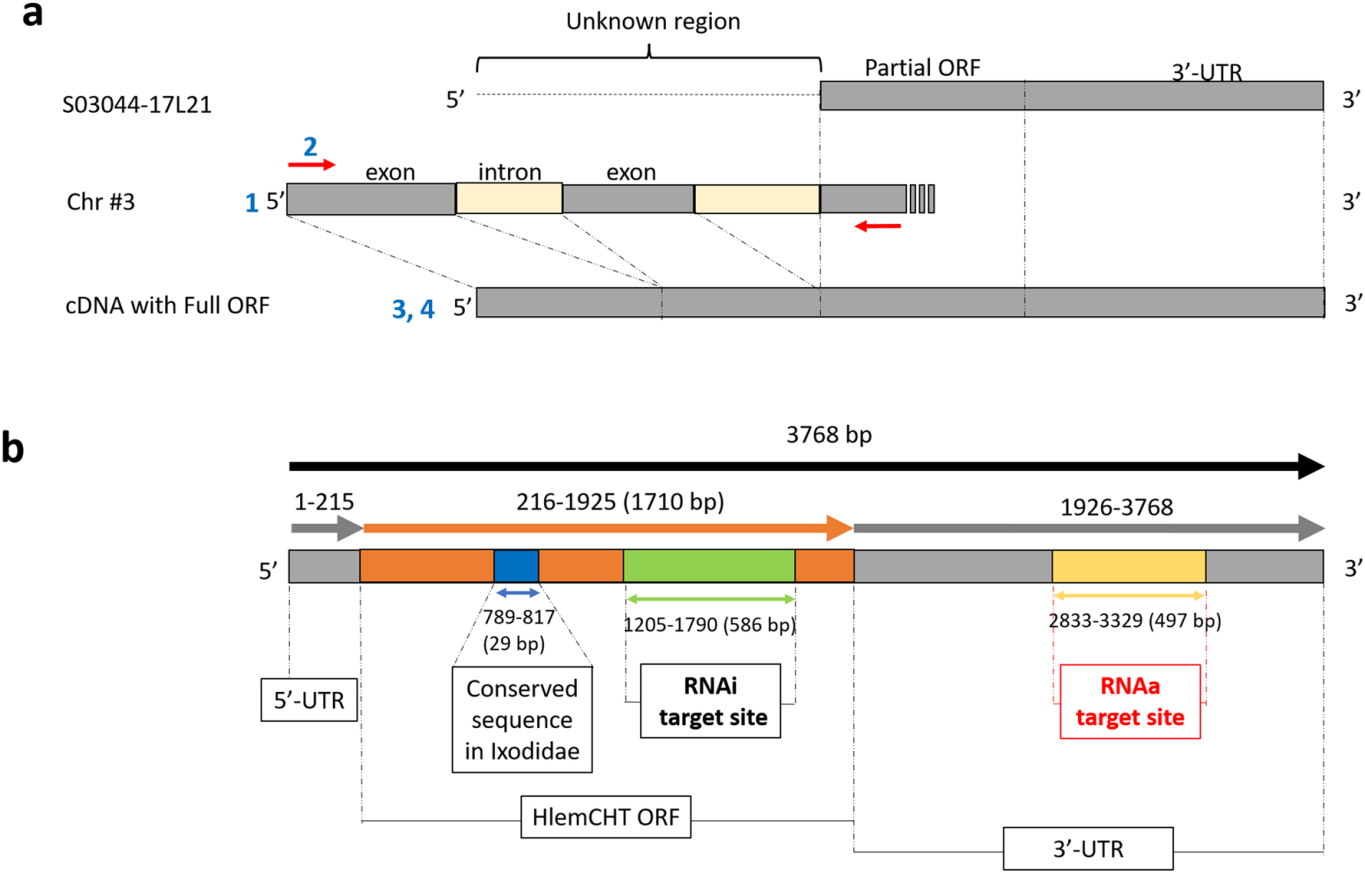
Full-length ORF of HlemCHT cDNA. (**a**) Strategy for obtaining full-length ORF of HlemCHT. (1) Predicted exons and introns on the upstream sequence of the putative endochitinase-like gene (S03044-17L21 identified in the cDNA library, namely, HlemCHT) were retrieved from Chromosome 3 (Chr #3) after a nucleotide BLAST search of a partial ORF sequence against both local and NCBI nr databases of *Haemaphysalis longicornis* genome*.* (2) Forward and reverse primers (Red arrows) were designed to target sequences before the predicted starting methionine, and sequences at about 150 bp downstream from the end of the partial ORF fragment respectively. (3) Amplified cDNA was sequenced after cloning into a vector. (4) Assembly of newly sequenced 5ʹ-cDNA fragment and already existing cDNA fragment into a contig sequence was done using CAP3 Sequence Assembly Program to obtain the full-length ORF. (**b**) HlemCHT gene structure showing the positions the RNAi target site (Green) within the full-length ORF (Orange) and the RNAa target site (Yellow) at the 3′-UTR. Also, within the ORF is a conserved sequence (Blue) in other ixodid tick species’ endochitinase-like genes.

To obtain the full-length open reading frame (ORF) of the putative endochitinase-like gene (S03044-17L21 identified in the cDNA library, namely, HlemCHT), we employed the strategy summarised in Fig. 1a. Briefly, predicted exons and introns on the upstream sequence of the HlemCHT gene were retrieved from Chromosome 3 after a nucleotide BLAST search of a partial ORF sequence against both local (using BioEdit 7.2) and National Center for Biotechnology Information (NCBI) non-redundant (nr) protein databases of *Haemaphysalis longicornis* genome (GenBank accession number JABSTR000000000)*.* Forward and reverse primers (Red arrows) were then designed to target sequences before the predicted starting methionine, and sequences at about 150 bp downstream from the end of the partial ORF fragment respectively. Amplified cDNA was sequenced after cloning into a vector and the assembly of newly sequenced 5ʹ-cDNA fragment and already existing cDNA fragment into a contig sequence was done using CAP3 Sequence Assembly Program to obtain the full-length ORF. We finally obtained a cDNA of full length, 3768 bp (Fig. 1b), with an ORF of 1710 bp long. Analysis using the Compute pI/MW function of the Expasy web tool (www.expasy.org/resources/compute-pi-mw) revealed that the ORF encoded 569 amino acid polypeptides, with a predicted molecular weight of 64.7 kDa. The 5ʹ- and 3ʹ- UTRs of the cDNA consisted of 215 bp and 1843 bp respectively (Fig. 1b). The full-length ORF sequences obtained in this study have been deposited in the DDBJ, EMBL, and GenBank databases. (Accession number LC744416).

BLAST analysis using the nucleotide BLAST (BLASTn) program on the web tool (https://blast.ncbi.nlm.nih.gov/Blast.cgi?PROGRAM=blastp&PAGE_TYPE=BlastSearch&LINK_LOC=blasthome) with HlemCHT (ORF) sequence as a query, showed homologs of HlemCHT in other tick species, such as *Rhipicephalus sanguineus* (Accession No. XP_049270163.1), *Rhipicephalus microplus* (Accession No. XP_037285628.1), *Dermacentor andersoni* (Accession No. XP_050044043.1), *Dermacentor silvarum* (Accession No. KAH7965576.1) and *Ixodes scapularis* (Accession No. XP_040067710.3) with sequence similarities of 88.10%, 89.35%, 87.64%, 88.66%, 88.05%, and 91.60% respectively. HlemCHT was also observed to have a conserved motif, TTCGACGGCTTCGACCTGGACTGGGAGTA. This motif was predicted by the web program Interpro scan (https://www.ebi.ac.uk/interpro/result/InterProScan/iprscan5-R20221202-102605-0383-52842065-p1m/) to code for an active site, characteristic of the protein family of glycoside hydrolases (Glycosyl hydrolases family 18 (GH18)) of which endochitinases belong.

### Expression profile of HlemCHT transcripts in different life cycle and feeding stages

**Figure 2:**
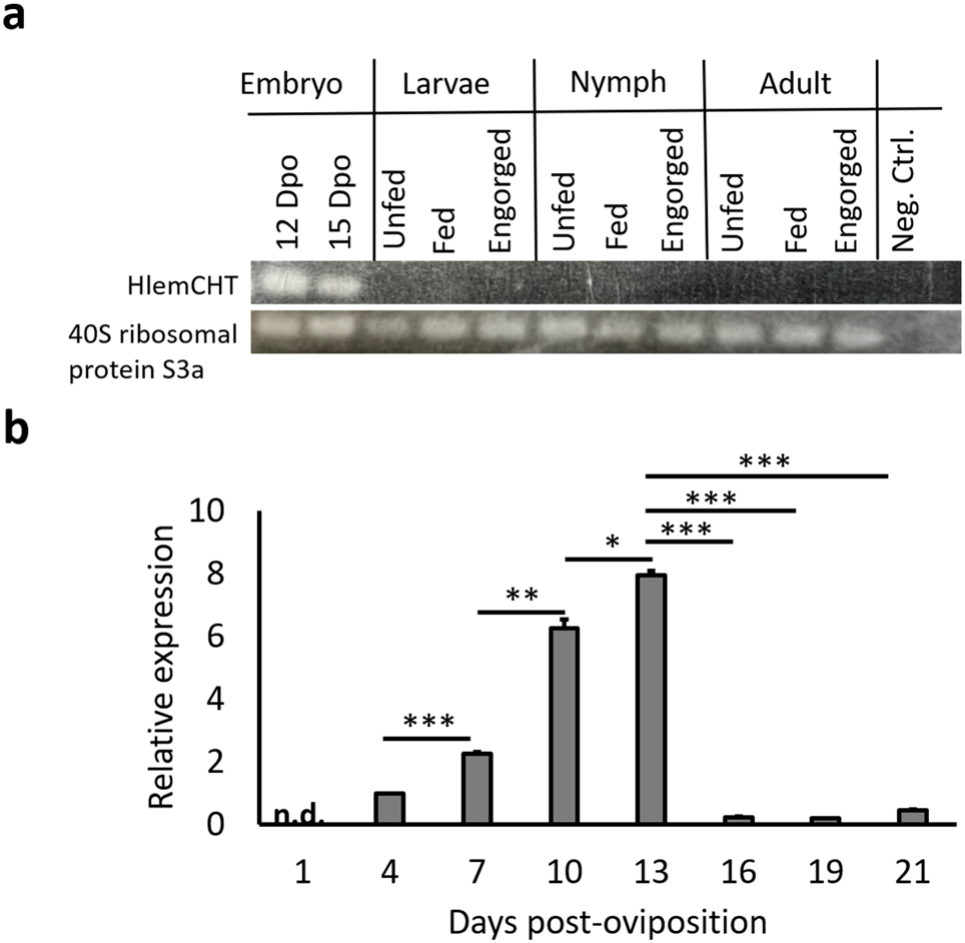
Normal expression of HlemCHT in *H. longicornis*. (**a**) RT-PCR analysis of HlemCHT in different developmental stages of *H. longicornis.* Analysis was performed using total RNA from eggs (12 and 15 Dpo), the whole body of larvae (unfed, partially fed, engorged), nymph (unfed, partially fed, engorged), and adult (unfed, partially fed, engorged). *H. longicornis* 40S ribosomal protein S3a was used as an internal control. PCR products were analysed on 1.5% agarose gel and stained with ethidium bromide for viewing. Original gels are presented in Supplementary Fig. S1. (**b**) RT-qPCR analysis of HlemCHT during embryogenesis of tick eggs. The expression of each mRNA was normalized to the expression of 40S ribosomal protein S3a measured in same RNA preparation. The relative expression values are represented as “means ± SD”. Error bars represent the SEM of 4 replicates. *Indicates a difference at P < 0.05; **designates P < 0.005; ***designates P < 0.001. The results shown are from a single experiment and are representative of three independent experiments. *n.d.* not determined.

To determine the normal expression of HlemCHT gene within the life cycle of *H. longicornis,* we investigated its expression in the different life stages (12/15 days embryonated eggs, larvae. nymphs and adults) of *H. longicornis* parthenogenetic ticks at different feeding stages (unfed, partially fed and fully engorged) using reverse-transcription PCR (RT-PCR)*.* The statutory expressed *H. longicornis* 40S Ribosomal S3a protein was used as an internal control gene, while a no template control served as a negative control. In our results, we observed HlemCHT to be solely present in the 12 and 15 dpo eggs (Fig. 2a), suggesting that the expression of HlemCHT is embryo-specific. In addition, we investigated the expression profile of HlemCHT in tick eggs during embryogenesis. To do this, eggs from fully-engorged maternal ticks were divided into eight (1-, 4-, 7-, 10-, 13-, 16-, 19- and 21-days post-infection) groups, according to which, they were harvested and frozen. Total RNA was then extracted from each group of tick eggs (50–100 /group) and quantitative reverse-transcription PCR (RT-qPCR) analysis was performed. *H. longicornis* 40S ribosomal gene S3a was used to as a reference gene to normalize the HlemCHT gene expression data. From our results, the relative expression of HlemCHT increased significantly (P < 0.005) with embryo development until day 13 dpo, which recorded the highest expression (Fig. 2b). Relative expression values per the days assessed are as follows: day 1 = no expression determined, day 4 = 1.00 ± 0.00, day 7 = 2.27 ± 0.04, day 10 = 6.25 ± 0.27 and day 13 = 7.95 ± 0.16. Beyond 13 dpo, the relative expression sharply declined as follows: day 16 = 0.24 ± 0.01, day 19 = 0.19 ± 0.02 and day 21 = 0.46 ± 0.03, suggesting that HlemCHT is highly expressed in *H. longicornis* eggs during mid-phase (day 12–15 dpo) embryogenesis.

### Induction of RNAa in *H. longicornis* using dsRNA

**Figure 3:**
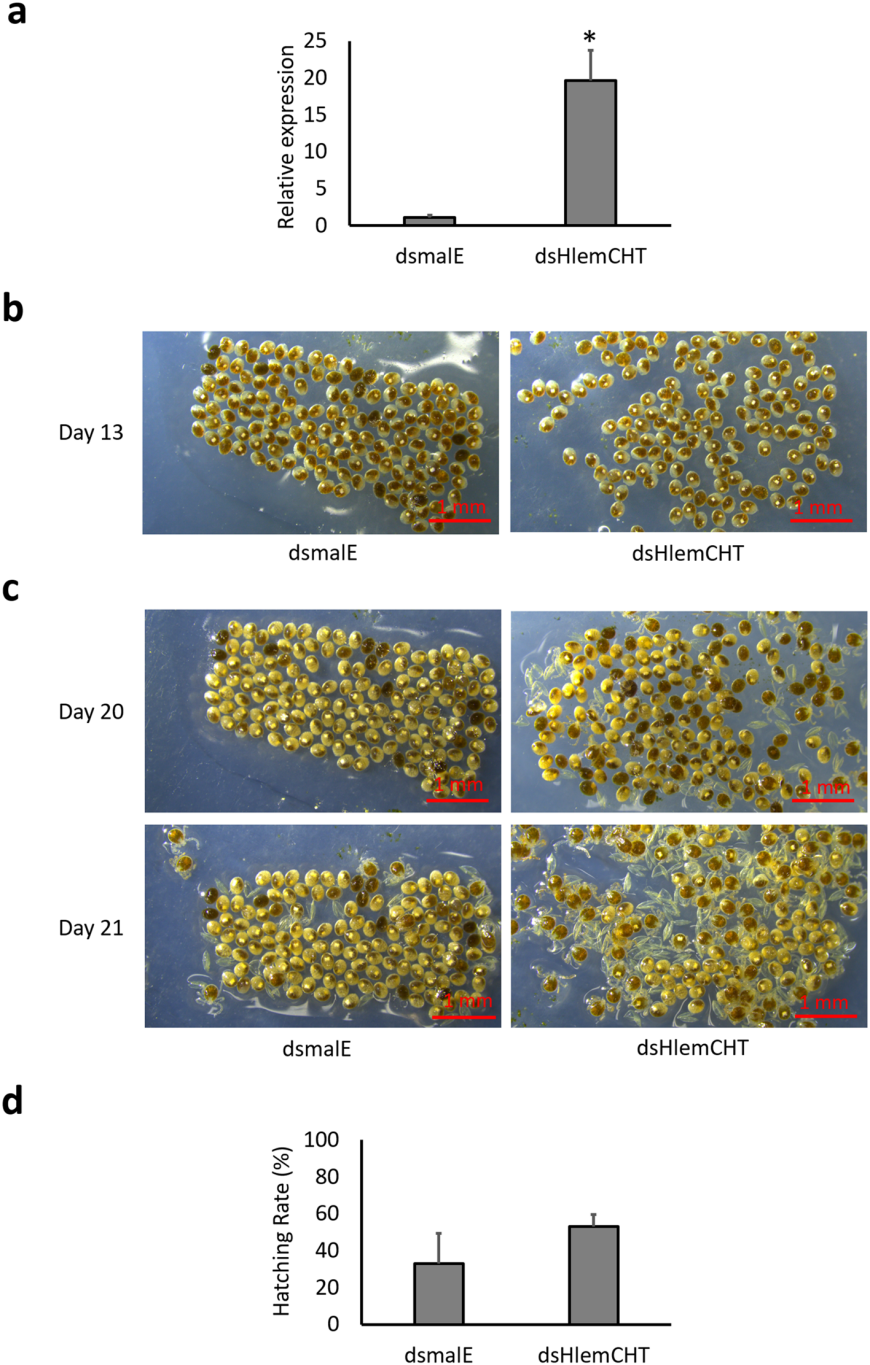
Confirmation of HlemCHT activation by dsRNA targeting the 3ʹ-UTR. (**a**) Relative gene expression of HlemCHT at 13 days post-oviposition (dpo). Data is represented by a histogram. Relative expression of the *H. longicornis* 40S ribosomal gene was used to normalize the HlemCHT gene expression data. Error bars represent the SEM of 4 replicates. *P = 0.0145. (**b**) Representative stereomicroscopic images of dsRNA-derived eggs at 13 dpo. (**c**) Representative stereomicroscopic images of dsRNA-derived eggs hatching at 20 and 21 dpo. (**d**) Histogram showing the comparison of the hatching rate of dsHlemCHT-derived eggs, as against eggs of dsEc-malE at 21 dpo. Error bars represent the SEM of 4 replicates. The results shown are from a single experiment and are representative of three independent experiments.

**Figure 4:**
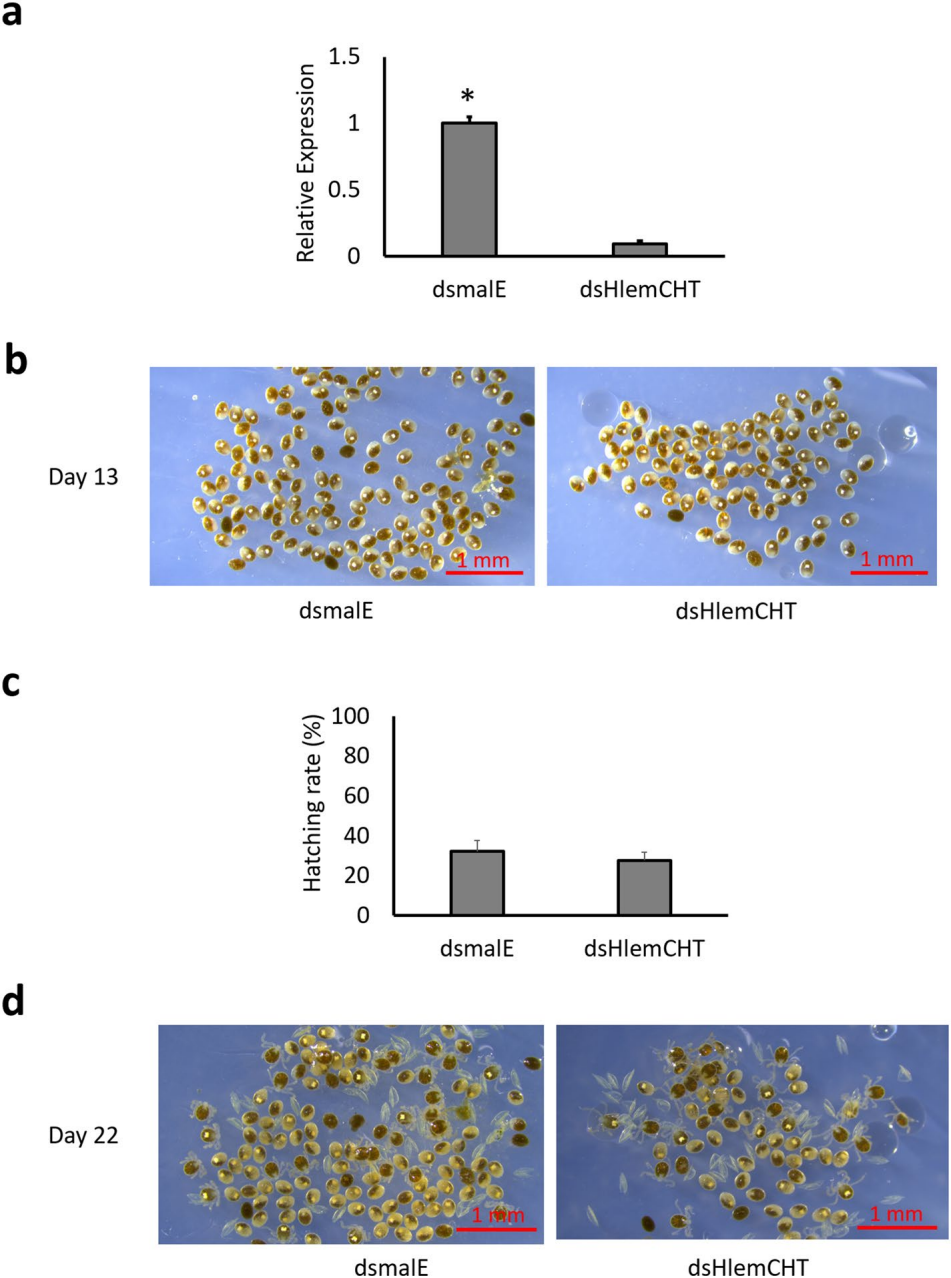
Confirmation of HlemCHT suppression by dsRNA targeting the ORF. (**a**) Relative gene expression of HlemCHT at 13 days post-oviposition (dpo). Data is represented by a histogram. Relative expression of the *H. longicornis* 40S ribosomal gene S3a was used to normalize the HlemCHT gene expression data. Error bars represent the SEM of 4 replicates. *P = 0.0001. (**b**) Representative stereomicroscopic images of dsRNA-injected tick eggs at 13 dpo. (**c**) Histogram showing the comparison of the hatching rate of dsHlemCHT-derived eggs, as against eggs of dsEc-malE at 22 dpo. Error bars represent the SEM of 4 replicates (**d**) Representative stereomicroscopic images of dsRNA-derived eggs hatching at 22 dpo. The results shown are from a single experiment and are representative of three independent experiments.

To investigate the potential occurrence of the RNAa phenomenon in eggs of *H. longicornis,* we targeted the 3ʹ-UTR of the HlemCHT gene because, among the many observations made concerning the genomic target site of saRNA, some have suggested that saRNAs demonstrate optimal function when they target sequences downstream from the 3′-UTR of the intended target gene^16^. Therefore, adult female ticks were injected with synthesized dsRNAs targeting either the 3ʹ-UTR of HlemCHT gene (dsHlemCHT) or a non-target control, *Escherichia coli* malE (Ec-malE) gene (dsEc-malE). RNA was then extracted from day 13 eggs derived from the dsRNA-injected maternal ticks because HlemCHT was observed to be highly expressed in tick eggs at 13 dpo (Fig. 2b), and as such, any changes in expression pattern, be it activation or suppression, would be clearly shown. The expression of HlemCHT was quantified by RT-qPCR analysis. In our results, targeting the 3ʹ-UTR, showed a 19 ± 8.20—fold significant (P = 0.0145) increase in HlemCHT expression in dsHlemCHT-derived eggs relative to dsEc-malE-derived eggs (Fig. 3a). In addition, morphological examination of the dsHlemCHT-derived eggs at 13 dpo, showed a normal phenotype characteristic of stage 12 embryo described in previous studies^44,45^ with evidence of limb formation, whitish strands of malpighian tubules and a distinctly formed bright white hindgut, similar to dsEc-malE-derived eggs (Fig. 3b). Moreover, dsHlemCHT- derived eggs were observed to begin hatching within 20 days post-oviposition, about 24 h earlier than dsEc-malE -derived eggs (Fig. 3c). Furthermore, at 21 days post-oviposition, when dsEc-malE-derived eggs began hatching, the hatching rate was 32.9 ± 32.6%. In contrast, the hatching rate for dsHlemCHT-derived eggs was relatively higher at 52.95 ± 13.50%, although not significant (P = 0.5066) (Fig. 3c,d). On the other hand, when the ORF of the same gene was targeted, the relative expression of HlemCHT was observed to be 0.09 ± 0.02 in dsHlemCHT-derived eggs as opposed to 1.00 ± 0.04 in dsEc-malE-derived eggs, suggesting a significant (P = 0.0001) downregulation of the gene in dsHlemCHT-derived eggs at 13 dpo (Fig. 4a). Interestingly, phenotypic observation of day 13 dsHlemCHT-derived eggs also revealed a normal phenotype as observed when the 3ʹ-UTR was targeted, with no difference observed between the dsEc-malE-derived and dsHlemCHT-derived eggs (Fig. 4b). Consequently, hatching of dsRNA-derived eggs begun on day 22 post-oviposition, with no significant (P = 0.5306) difference observed between hatching rates of dsHlemCHT-derived eggs (27.73 ± 4.00%) and dsEc-malE-derived eggs (32.26 ± 5.51%) (Fig. 4c,d). These results, therefore, indicate the importance of the 3ʹ-UTR as an essential target for dsRNA-mediated activation of the HlemCHT gene in tick eggs.

## Discussion

RNAa is the targeted upregulation of a specific gene^21^. The use of RNAa was limited to only a few major mammalian models^3,11,13,46^, but it has now been reported in other organisms such as plants, bacteria, *C. elegans,* and recently, mosquito arthropods. It is currently being explored as a potential tool for the specific activation of genes, towards the development of novel therapeutics for undruggable disease and vector control^14,17^.

In the present study, we targeted the 3ʹ-UTR of a novel endochitinase-like gene (HlemCHT), in *H. longicornis* eggs for dsRNA-mediated gene activation and showed an increased expression of the gene on day 13 post-oviposition. In a simultaneous experiment, when we targeted sequences within the ORF of the same gene, we observed a downregulation of the gene (Fig. 4), suggesting the possibility of a dsRNA-mediated gene activation, which may be based on the association of the dsRNA (saRNA) with the 3ʹ-UTR of the gene.

Different dsRNA-mediated gene activation mechanisms have been proposed to involve the 3ʹ-UTR. For instance, it has been suggested that the saRNAs that target the 3ʹ-UTR trigger a looping mechanism bringing the 3′-terminus and promoter into proximity to allow the recruitment of additional proteins and modulation of promoter activity^16^. It has also been proposed that saRNA molecules may target AU-rich elements in 3ʹ-UTR of mRNA molecules and induce gene expression at the transcriptional level. For example, two studies showed miR-369-3 to positively regulate mRNA translation by targeting AU-rich elements in 3′-UTRs in stressed cells^47^. However, in this study, characteristic AU-rich elements were not found in the 3′-UTR of HlemCHT, which suggests that the gene activation observed in our study may probably be due to a looping mechanism triggered by saRNAs targeting the 3′-UTR, and bringing the 3′ terminus and promoter into proximity for the activation of the gene, leading to a 19-fold increase in gene expression. Further studies are however required to elucidate the exact mechanism of RNAa in ticks.

This study also established a positive relationship between dsRNA-mediated gene expression and egg hatching, concerning hatching start time and percentage of hatched eggs. We observed that eggs of dsHlemCHT began hatching about 24 h earlier than control eggs, as well as a slightly higher percentage of hatched eggs by 21 dpo, suggesting that the possible dsRNA-mediated gene activation of HlemCHT observed on day 13, drove the early hatch of dsHlemCHT -derived eggs. Indeed, several reports on the role of chitinase in eggs of arthropods and nematodes, have indicated that chitinase (-like) genes are vital for successful egg development and hatching^48–53^. For example, knockdown of *Tribolium* (flour beetle) chitinase-like gene, TcCHT10 in adult females, resulted in none of the eggs hatching, despite the presence of fully developed embryos in these eggs^52^. In our study, a single injection of dsRNA targeting the ORF, into unfed adult female ticks resulted in no phenotypic changes, despite the downregulation of the HlemCHT gene observed (Fig. 4). However, a repeated introduction (once daily for 3 consecutive days) of dsRNA into fully-engorged adult female ticks before the start of oviposition resulted in a downregulation of the gene and the subsequent delay or reduction of hatching rate of *H. longicornis* eggs. The results for this modified RNAi method will be reported in another article being prepared currently.

Overall, this study's results provide some evidence of the presence of RNAa phenomenon in the tick vector, *H. longicornis* for the first time. More studies are however required to provide further insight into the detailed mechanisms by which the expression of genes is activated by dsRNA (saRNAs) in ticks. Currently, no established gene manipulation technology permits stable gene knock-ins for applications such as gene replacement and over-expression in ticks^41^. Therefore, the discovery of RNAa phenomenon in ticks has the potential to provide new technology for the application of RNAa in the over-expression of both endogenous and exogenous genes in ticks. For this purpose, we will investigate other *H. longicornis* genes for the established phenomenon of RNAa in subsequent studies. Furthermore, reports of RNAa in *Ae. aegypti*^17^ and *H. longicornis* (this study), show the potential of saRNAs^40^, considering their small size, versatility, and safety, to reverse phenomena, such as the emergence of acaricide/insecticide resistance due to the downregulation of critical genes^54,55^. Finally, this report will hopefully provide new opportunities, based on the usefulness of RNAa as a tool for future research in arthropod biology, to modulate gene responses, to restrict the expansion of tick populations and transmission of pathogens to reduce the global burden of ticks and tick-borne diseases.

## Materials and methods

### Ticks and animals

The parthenogenetic Okayama strain of the ixodid tick *H. longicornis* bred by feeding on mice as described previously^56^, was maintained at the Department of Parasitic and Tropical Medicine, Kitasato University School of Medicine (KUSM), Sagamihara, Kanagawa, Japan. Its ability to reproduce parthenogenetically by feeding on the blood of several hosts including mice makes it easier to maintain in the lab. Therefore has become an ideal model tick species for tick experiments in many labs globally as can be seen in various publications^27,37,57–66^. Fifteen-week-old BALB/c CrSlc mice (SLC Japan, Shizuoka, Japan) were adapted to standard animal husbandry conditions (25 °C, 60% Relative Humidity (RH)) for 7 days, and subsequently to tick maintenance condition (25 °C, 70% RH) for 2 days before the experiment.

### Ethical statement

Mice maintenance and tick feeding experiments were carried out under protocols approved by the Animal Care and Use Committee, KUSM (Approval no. 2022.073), which are based on the Law for the Humane Treatment and Management of Animals, Standards Relating to the Care and Management of Laboratory Animals Relief of Pain (the Ministry of the Environment), Fundamental Guidelines for Proper Conduct of Animal Experiment Related Activities in Academic Research Institutions (the Ministry of Education, Culture, Sports, Science Technology), as well as, the Guidelines for Proper Conduct of Animal Experiments (the Science Council of Japan), and are reported in accordance with the ARRIVE guidelines^67,68^. At the end of the experiments, the animals were euthanized by exsanguination under anesthesia with isoflurane followed by cervical dislocation.

### Cloning and sequencing of full-length HlemCHT

The plasmid (pCR-Blunt II-TOPO/S03044-17L21) containing partial cDNA encoding the HlemCHT transcript was first isolated from a previously established cDNA library^43^. A BLASTn search of the partial sequence of the 5ʹ-terminal (ATGACGTATGATCTGCGTGGGAACTGGGCTGGCTTTACGG) of this cDNA was performed locally against *Haemaphysalis longicornis* genome database (GenBank accession number JABSTR000000000) with the p-value of < 10^–6^ by using BioEdit 7.2 (https://bioedit.software.informer.com/7.2/). According to this analysis, the partial sequence was identified in Chromosome No.3 with an expected value of 1.00E−12 and an identity of 97%. By using this partial genome sequence, the BLASTN program on the web tool (https://blast.ncbi.nlm.nih.gov/Blast.cgi?PAGE_TYPE=BlastSearch) was done again against the NCBI nr database and the predicted HlemCHT gene (GenBank accession number currently not available due to ongoing updates of genome annotations) located on the chromosome No. 3 of *H. longicornis* was identified. According to the previous (11th February 2022) annotated information, the upstream sequence including predicted exons and introns was retrieved to design the forward primer on the most upstream region before starting methionine. Reverse primer was also designed at about 150 bp downstream of the edge of known cDNA fragment. Both forward and reverse primers on the 5ʹ-UTR were designed to amplify the unknown fragment flanked by the starting methionine and the partial cDNA sequence. Primers used in this study were purchased from Eurofins Genomics K.K., Tokyo, Japan, and are listed in Table 1. RT-PCR was then performed to amplify the unknown target sequence. The amplification was performed using a PCR program of 94 °C for 2 min, 35 cycles of 98 °C for 30 s, either 64–67 °C for 30 s, and 68 °C for 30 s, followed by elongation for 1 min at 68 °C. The PCR products were purified from agarose gel using VIOGENE Gel/PCR DNA isolation system Extraction Kit (VIOGENE BIOTEK, TAIWAN) according to the manufacturer’s instructions. Cloning of the target sequence into pCR-Blunt II-TOPO vector was also done according to the manufacturer’s instruction and transformed into DH5α *E. coli* competent cells (Nippon gene DO., LTD., Tokyo, Japan). Positive colonies were sub-cultured and plasmid purification was done using PureLink™ Quick Plasmid Miniprep Kit (Invitrogen by ThermoFisher Scientific K.K., Tokyo, Japan), after which samples were sequenced by Sanger technology in an independent external company (Eurofins Genomics K.K., Tokyo, Japan). Starting methionine and 5’UTR sequence were confirmed accordingly. The newly sequenced 5ʹ-cDNA fragment and already existing cDNA fragment were assembled into a contig sequence using CAP3 Sequence Assembly Program on the web tool (https://doua.prabi.fr/software/cap3)^69^. Full-length ORF predicted to encode this novel endochitinase-like protein was isolated and confirmed from another set of RT-PCR with the Full HlemCHT ORF primer set (Table 1) and sequence analysis at the company (Eurofins Genomics K.K., Tokyo, Japan). The strategy for cloning and sequencing of full-length HlemCHT is illustrated in Fig. 1a.

**Table 1:** List of primers.

Primers	Position	Primer sequence	Tm ( °C)
HlemCHT 5'-complete ORF	Forward	TGTATGCGTTCAGTGCTACCGGGCGTA	65.1
	Reverse	TAAAGCCAGCCCAGTTCCCACGCAGAT	65.1
HlemCHT 5 '-TOPO colony PCR	Forward	TGGTTGGGCTGAGGGCGGAAAGAAGTA	65.1
	Reverse	TGTAGACGAAACTTGGCGACGGGCACA	65.1
HlemCHT 5'-complete ORF sequencing	Forward	TGGTTGGGCTGAGGGCGGAAAGAAGTA	65.1
	Reverse	TGTAGACGAAACTTGGCGACGGGCACA	65.1
Full HlemCHT ORF	Forward	ACGAGCTGTGCGTGCTTGCCAAAGTCT	65.1
	Reverse	TGCAGGCTAGTTTTTCCGGCTAGCCACTC	66.2
Full HlemCHT ORF sequencing	Forward	CGGGGAGGTGCATACTCTGACAGGGCTAA	67.6
	Reverse	CGGTGTTGCCCTTGTCACTCAGCGTGT	66.6
HlemCHT (3ʹ-UTR) dsRNA synthesis (T7)	Forward	*gaaattaatacgactcactataggTCACATGCTCACGATCACCCGCATACC	65.1
	Reverse	*gaaattaatacgactcactataggGGACGTGTCCTTGCACGTTGGCATACA	65.1
HlemCHT (3ʹ-UTR) dsRNA synthesis (Gene-specific)	Forward	TCACATGCTCACGATCACCCGCATACC	65.1
	Reverse	GGACGTGTCCTTGCACGTTGGCATACA	65.1
HlemCHT (ORF) dsRNA synthesis (T7)	Forward	*gaaattaatacgactcactataggCAAGGGCAACACCGGACTCAGGGCTTA	66.6
	Reverse	*gaaattaatacgactcactataggGAACATCACCATGGGCGTCCCGTACAC	66.6
HlemCHT (ORF) dsRNA synthesis (Gene-specific)	Forward	CAAGGGCAACACCGGACTCAGGGCTTA	66.6
	Reverse	GAACATCACCATGGGCGTCCCGTACAC	66.6
MalE gene	Forward	CGACCGCTTTGGTGGCTACGCTCAATC	66.6
	Reverse	CCGCAGTACGCACGGCATACCAGAAAG	66.6
MalE dsRNA synthesis (T7)	Forward	*gaaattaatacgactcactataggGTTGGCTGAAATCACCCCGGACAAAGC	65.1
	Reverse	*gaaattaatacgactcactataggTTAATACCTGCGCTCAGCACGCCAACG	65.1
MalE dsRNA synthesis (Gene-specific)	Forward	GTTGGCTGAAATCACCCCGGACAAAGC	65.1
	Reverse	TTAATACCTGCGCTCAGCACGCCAACG	65.1
Full HlemCHT ORF-TOPO colony PCR / HlemCHT RT-qPCR	Forward	CATACCGCACGAGAACGACTGCACCAA	65.1
	Reverse	CACAGTTGCCGTTGTCCTGGTTCCACA	65.1
40S ribosomal S3a RT-qPCR	Forward	CCTCATCCGCAAGGTGAAGGTGCTCAA	65.1
	Reverse	AAGCGCCCTCAGACATTCTCCAGCACA	65.1

*gaaattaatacgactcactatagg—T7 promoter sequences.

### Expression pattern of HlemCHT in different life cycle and feeding stages

To determine the normal expression of HlemCHT in different developmental and feeding stages of *H. longicornis,* reverse transcription-polymerase chain reaction (RT-PCR) was employed. To do that, larval (150), nymphal(100) and adult (7) stages of *H. longicornis* ticks were allowed to feed on the shaven back of BALB/c mice (3 mice per each developmental stage). At the beginning of the tick expansion period (3–5 days after tick attachment), About 50 partially engorged larvae, 50 partially engorged nymphs and 5 partially engorged adult female ticks, were detached carefully with fine tip forceps without breaking the hypostome (in the case of the adults) or gently passing a soft-bristled paint brush over the attached ticks to detach them (in the case of larvae and nymphs). The rest of the ticks were allowed to fully engorge and drop off by themselves. Two fully engorged adult ticks were allowed to lay eggs under the suitable temperature of 25 °C and > 70% humidity. Eggs laid by each engorged tick were further divided into two groups (50–100/group) and incubated for either 12 or 15 days post oviposition. Either tick developmental stages (unfed, partially fed and fully engorged (after egg laying)) or egg samples collected at their respective times were immediately flash frozen in liquid nitrogen and stored at − 80 °C until used. For RNA extraction, frozen samples were ground to a fine powder, using a mortar and pestle kept in liquid nitrogen. Total RNA was isolated from the resulting powder with the RNeasy mini kit (Qiagen, Valencia, CA, USA) according to the manufacturer’s instructions. The quantity and integrity of the RNA was measured spectrophotometrically by ultraviolet light absorbance using NanoDrop™ One Microvolume UV–Vis Spectrophotometer (Thermo Scientific, Rockford, IL, USA) and stored at − 80 °C until use. Complementary DNA (cDNA) was synthesized from about 150 ng total RNA extracted from the tick/egg samples using ReverTrace™ qPCR RT Master Mix with gDNA remover (Toyobo, Japan) kit according to manufacturer’s instructions. Polymerace chain reactions (PCRs) were performed to amplify a 110 bp region of HlemCHT and a 192 bp region of 40S Ribosomal protein S3a using primers purchased from Eurofins Genomics K.K., Tokyo, Japan. Primers are shown in Table 1. The reaction mixtures for PCR were prepared using the KOD Plus Neo ™ PCR kit (Toyobo, Japan) and band amplification was done using a Bio-Rad T100™ Thermal Cycler, with the following cycling conditions; initial denaturation at 94 °C for 2 min, followed by 35 cycles of denaturation at 98 °C for 10 secs, annealing at 66 °C for 30 secs (for both genes) and extension at 68 °C for 10 secs, with a final extension performed at 68 °C for 1 min. The PCR products were separated by a 1.5% agarose gel electrophoresis and stained with ethidium bromide for viewing.

### Expression pattern of HlemCHT in tick eggs during embryo development

By the same method of blood feeding and egg laying described above, eggs were collected from two different fully engorged maternal ticks, and each set was divided into eight (1-, 4-, 7-, 10-, 13-, 16-, 19- and 21-days post-infection) groups, according to which, eggs were harvested and frozen. Total RNA from tick eggs (about 50–100/group) was extracted, followed by subsequent cDNA synthesis as have already been described in this study. RT-qPCR analysis was then performed using TB Green® Premix DimerEraser™ kit (Takara, Japan) according to the manufacturer’s instructions with the same primer sets (Table 1) already described for either HlemCHT or *H. longicornis* 40 s ribosomal protein S3a to determine their expression levels. Each experiment was done with two different RNA samples per each group and were run in duplicates. The experiment was repeated three times, and the data were analyzed with the 2^−ΔΔCt^ method^70^ using Microsoft Excel version 2019 and expressed as mean ± standard deviation. Statistical significance was determined using an unpaired two-tailed student’s t-test, with a value of P < 0.05 considered significant after the normal distribution and homogeneity of variance were confirmed by Kolmogorov–Smirnov and F-tests respectively.

### Double-stranded RNA (dsRNA) synthesis

To investigate the occurrence of RNAa in ticks, the 3ʹ-UTR of the HlemCHT gene was targeted. A template partial-length cDNA fragment at the 3ʹ-UTR of the HlemCHT transcript (497 bp) was therefore amplified from a pCR-Blunt II-TOPO/S03044-17L21 plasmid by PCR, using primer sets flanked by a T7 promoter and gene-specific primer sets (Table 1) which were designed using Primer 3 (v. 0.4.0) program (https://bioinfo.ut.ee/primer3-0.4.0/) and purchased from Eurofins Genomics K.K., Tokyo, Japan. To provide further evidence that the association of dsRNA to the 3ʹ-UTR is vital for the occurrence of RNAa, we also synthesized dsRNA targeting the ORF in a parallel experiment. A template partial-length cDNA fragment within the ORF of the HlemCHT transcript (586 bp) was therefore amplified from the same plasmid using T7 promoter-flanked primers, as well as gene-specific primer sets designed and purchased as already described (Table 1). As a negative control (also a non-target control), dsRNA targeting *Escherichia coli* malE (Ec-malE) was also synthesized. Ec-malE is absent in ticks and dsRNA targeting Ec-malE has been shown in several studies^61–63,71^ not to affect normal tick physiology, including egg laying and the normal expression of tick-specific genes. Moreover, it has been shown to give comparable results to a no injection (non-manipulated) control in most of the studies referred to above. Briefly, the Ec-malE DNA fragment (848 bp) was first amplified from *E. coli* using primers (Table 1) designed and purchased as already described. The amplified fragments were then cloned into the pCR-Blunt II-TOPO vector as earlier described. Positive colonies were sub-cultured and plasmid purification was also done as already described. A template DNA fragment of the Ec-malE gene (577 bp) was then amplified from the pCR-Blunt II-TOPO/malE plasmid by PCR, using T7 promoter-flanked primers, as well as gene-specific primer sets designed and purchased as already described (Table 1). The amplification was performed using a PCR program of 94 °C for 2 min, 35 cycles of 98 °C for 30 s, either 66 °C (HlemCHT/3ʹ-UTR), 66 °C (HlemCHT/ORF) or 67 °C (Ec-malE) for 30 s, and 68 °C for 30 s, followed by elongation for 1 min at 68 °C. The PCR products were purified from agarose gel using VIOGENE Gel/PCR DNA isolation system Extraction Kit (VIOGENE BIOTEK, TAIWAN) according to the manufacturer’s instructions. DsRNAs of HlemCHT (3ʹ-UTR), HlemCHT (ORF) and Ec-malE were synthesized by in vitro transcription using the T7 RiboMAX™ Express RNAi System (Promega, Madison, WI, USA) according to the manufacturer’s instructions. The purity of synthesized dsRNAs was checked by running 1 µL of diluted dsRNA (1:10, 1:100, and 1:1000 with Nuclease-Free water) in a 1.5% agarose gel electrophoresis. Concentrations of dsRNAs were also measured by ultraviolet light absorbance using NanoDrop™ One Microvolume UV–Vis Spectrophotometer (Thermo Scientific, Rockford, IL, USA).

### Microinjection of dsRNA into adult female ticks

A volume of 0.5 μl of 2 μg/μl either HlemCHT (3ʹ-UTR), HlemCHT (ORF) or Ec-malE dsRNA was injected into the hemocoel from the fourth left coxa of unfed adult female *H. longicornis* ticks (10 ticks per each treatment) with a glass needle generated by a micropipette PC-10 puller (Narishige International, NY, USA) using an IM-11-2 pneumatic microinjector (Narishige International, NY, USA). Injected ticks were left for 18 h at 25 °C in a moist chamber. After 18 h, the dsRNA-injected ticks (dsHlemCHT (3ʹ-UTR), dsHlemCHT (ORF) and dsEc-malE) were observed for any mortality due to traumatic injury resulting from injection. Live ticks within each treatment group were then placed on a mouse to blood feed as earlier described. After 3–4 days of tick attachment, partially fed ticks were removed, leaving 2 ticks on each mouse for optimal blood feeding and engorgement. Each engorged tick was allowed to lay eggs after detachment from the host mouse.

### Maintenance and monitoring of dsRNA-injected ticks’ eggs

Eggs laid daily by each of the engorged ticks were kept separately in the wells of 24-well plates under humidified conditions at 25 °C. Some of the egg samples were allowed to develop and hatch, while others were collected on day 13 post-oviposition for confirmation of either the activation or suppression of gene expression by dsRNAs. To monitor the embryonic development of eggs till larval hatching, 1% agarose in distilled water, with 2.5 µM amphotericin B (AmpB) was dispensed into 3.5 cm petri dishes and allowed to solidify. Eggs were placed on solidified gel and covered slightly with halocarbon oil. Petri dishes containing eggs were kept under humidified conditions at 25 °C. Tick eggs were monitored daily for any morphological changes under a stereomicroscope (Lecia microsystems, Wetzlar, Germany). The hatching rate was calculated as follows: (Number of hatched eggs / total number of eggs monitored) × 100%. The data were analyzed using Microsoft Excel version 2019 and expressed as mean ± standard deviation. Statistical significance was determined using an unpaired two-tailed student’s t-test, with a value of P < 0.05 considered significant after the normal distribution and homogeneity of variance were confirmed by Kolmogorov–Smirnov and F-tests respectively.

### Confirmation of dsRNA-induced gene activation (or silencing) in tick eggs.

To confirm the activation (or silencing) of HlemCHT by dsRNAs, 50–100 eggs from each of the two sets of samples per dsRNA-treatment group, were harvested and frozen on day 13 post-oviposition, after which RNA extraction, cDNA synthesis and RT-qPCR analysis were performed as previously described in this study. Each experiment was done with two different RNA samples per each group, which were run in duplicates. The experiments were repeated three times, and data were analyzed as previously described in this study.

## Supplementary Information

Supplementary Figure S1.

## Data Availability

The datasets used and/or analyzed during the current study are available from the corresponding author on reasonable request. Please note that the details regarding the EST database and cDNA library described in this experiment can be referred from Umemiya-Shirafuji et al.^43^.
