# Supplementary material for: RNA activation in ticks
Source: Sci Rep. 2023 Jun 8;13:9341. doi: 10.1038/s41598-023-36523-4 (PMC10250327; doi:10.1038/s41598-023-36523-4)
Supplement: Supplementary file 1 — Supplementary Figure S1. [file 41598_2023_36523_MOESM1_ESM.docx]

**Article**

**RNA activation in Ticks**

Kofi Dadzie Kwofie^1,2^, Emmanuel Pacia Hernandez^1,3^, Anisuzzaman^4^, Hayato Kawada^1,5^, Yuki Koike^5^, Sana Sasaki^5^, Takahiro Inoue^5^, Kei Jimbo^5^, Fusako Mikami^1^, Danielle Ladzekpo^2,6^, Rika Umemiya-Shirafuji^7^, Kayoko Yamaji^8^, Tetsuya Tanaka^9^, Makoto Matsubayashi^10^, Md Abdul Alim^4^, Samuel Kweku Dadzie^2^, Shiroh Iwanaga^11,12^, Naotoshi Tsuji^1,5^, Takeshi Hatta^1,5 *^

^1^ Department of Parasitology and Tropical Medicine, Kitasato University School of Medicine, Sagamihara, Kanagawa, 252-0374, Japan.

^2^ Department of Parasitology, Noguchi Memorial Institute for Medical Research, College of Health Sciences, University of Ghana, Legon, Accra P.O. Box LG 581, Ghana*.*

^3^ Department of Veterinary Paraclinical Sciences, College of Veterinary Medicine, University of the Philippines at Los Baños, College, Laguna, Philippines, 4031

^4^ Department of Parasitology, Faculty of Veterinary Science, Bangladesh Agricultural University, Mymensingh 2202, Bangladesh.

^5^ Department of Molecular and Cellular Parasitology, Graduate School of Medical Sciences, Kitasato University, Sagamihara, Kanagawa 252-0374, Japan.

^6^ Department of Environmental Parasitology, Tokyo Medical and Dental University, Bunkyo-Ku, Tokyo 113-8510, Japan.

^7^　National Research Center for Protozoan Diseases, Obihiro University of Agriculture and Veterinary Medicine, Obihiro, Hokkaido, 080-8555, Japan.

^8^　Department of Tropical Medicine and Center for Medical Entomology, The Jikei University School of Medicine, Minato-ku, Tokyo, 105-8461, Japan.

^9^　Laboratory of Infectious Diseases, Joint Faculty of Veterinary Medicine, Kagoshima University, Kagoshima 890-0065, Japan.

^10^ Department of Veterinary Immunology, Graduate School of Veterinary Sciences, Osaka Metropolitan University, Izumisano, Osaka 598-8531, Japan.

^11^ Department of Molecular Protozoology, Research Institute for Microbial Diseases, Osaka University, Yamadaoka, Suita, Osaka, Japan.

^12^ Center for Infectious Disease Education and Research (CIDER), Osaka University, Yamadaoka, Suita, Osaka, Japan.

***** Correspondence: htakeshi@med.kitasato-u.ac.jp; Tel.: +81-42-778-9312


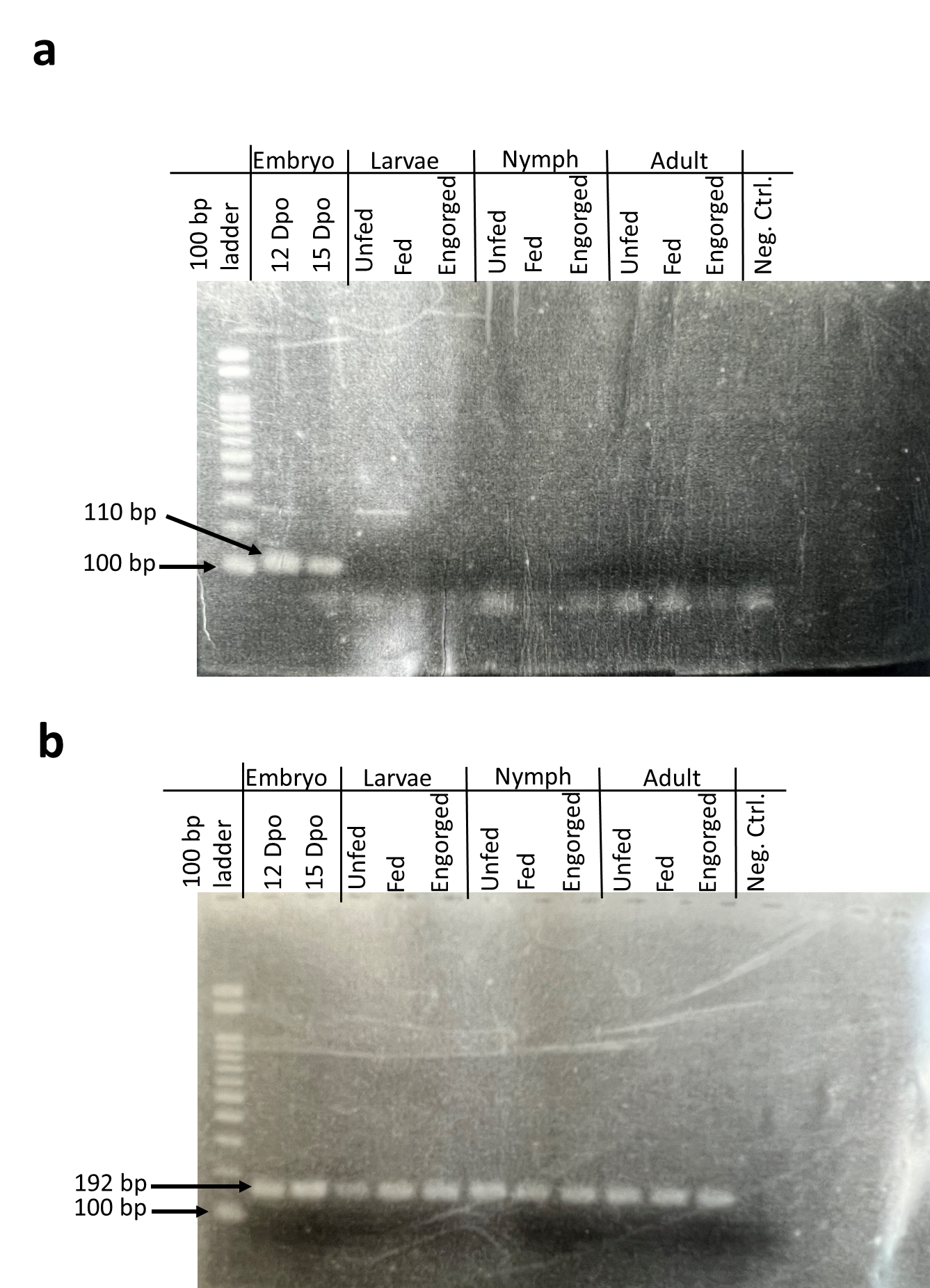


**Supplimentary figure S1**. **Expression of HlemCHT in different developmental stages of *H. longicornis****.* Original images of agarose gel electrophoresis after RT-PCR analysis showing amplified bands for **(a)** HlemCHT (110 bp) and **(b)** Internal control gene, *H. longicornis* 40S ribosomal protein S3a (192 bp). Analysis was performed using total RNA from eggs (12 and 15 days post-ovipostion (dpo)), the whole body of larvae (unfed, partially fed, engorged), nymph (unfed, partially fed, engorged), and adult (unfed, partially fed, engorged). PCR products were analysed on 1.5 % agarose gel and stained with ethidium bromide for viewing.
